# Evaluation of an Age-Friendly Health System: 4Ms Assessments and Outcomes in Hospitalized Older Adults

**DOI:** 10.1093/geroni/igaf122.3162

**Published:** 2025-12-31

**Authors:** Justin Lam, Susan Kwiatek, Shahidul Islam, Mark Tursi, Christian Nouryan, Philip Solomon, Maria Carney, Edith Burns

**Affiliations:** Donald and Barbara Zucker School of Medicine at Hofstra/Northwell, Brooklyn, New York, United States; Northwell Health, New Hyde Park, New York, United States; Northwell Health, New Hyde Park, New York, United States; Northwell Health, New Hyde Park, New York, United States; Northwell Health, Great Neck, New York, United States; Northwell Health, New Hyde Park, New York, United States; Zucker School of Medicine at Hofstra/Northwell, New Hyde Park, New York, United States; Zucker School of Medicine Hofstra-Northwell, Manhasset, New York, United States

## Abstract

The Institute for Healthcare Improvement developed the 4Ms framework (What Matters Most/Medication/Mentation/Mobility) to facilitate Age-Friendly Health Systems (AFHS). Each M has evidence-based screening tools leading to improved outcomes among older adults. We report screening 4Ms vs. 1-3Ms outcomes for hospitalized adults aged ≥65 across hospitals in a large healthcare system. Demographics and clinical data abstracted manually from EMR and from 4Ms AFHS dashboard.

**Population:**

Patients ≥65y/o admitted July-September 2024 to eight hospitals committed to care excellence. Outcomes: Length of stay (LOS), 30-day readmission, 30-day ED visits, mortality. Of 3,103 older adult admissions, 298 (9.6%) received all 4Ms and 2,805 received 1-3Ms. Median age: for 4Ms, 84 (IQR:76-90) and for 1-3Ms, 77 (IQR:71-84). Primary language was English for both. Median LOS: for 4Ms, 9 days (IQR:6-15) and for 1-3Ms, 9 days (IQR:5-16). Those receiving 4Ms had a lower AMPAC mobility score (13 vs 18, p < 0.001) fewer 30-day ED visits (16% vs. 22%, p = 0.02), higher mortality (11% vs. 5.3% p < 0.001), and trend towards lower 30-day readmissions (13% vs. 16%, p = 0.2). In conclusion, a small proportion of hospitalized older adults were screened for all 4Ms. Those with 4Ms were older with lower mobility. They had fewer post-discharge ED visits and a trend towards lower readmissions which may become significant with accrual of more events. Higher mortality may reflect age, greater illness burden. This preliminary assessment provides evidence for 4Ms care as a quality framework associated with key patient outcomes.

